# Diabetes related knowledge among residents and nurses: a multicenter study in Karachi, Pakistan

**DOI:** 10.1186/1472-6823-12-18

**Published:** 2012-09-11

**Authors:** Asma Ahmed, Abdul Jabbar, Lubna Zuberi, Muhammad Islam, Khusro Shamim

**Affiliations:** 1 Department of Medicine, The Aga Khan University Hospital, Karachi, Pakistan; 2 Crozer-Chester Medical Center, 1 Medical Center Blvd Ste 101, Chester, Pennsylvania, 19013, USA; 3 Department of Community Health Sciences, Aga Khan University, Karachi, Pakistan; 4 Department of Emergency Medicine, The Aga Khan University Hospital, Karachi, Pakistan

## Abstract

**Background:**

Assessment of knowledge among resident trainees and nurses is very important since majority of patients admitted in hospital have underlying diabetes which could lead to adverse clinical outcomes if not managed efficiently. Therefore, the purpose of this study was to evaluate and compare the knowledge related to the management of diabetes among registered nurses (RN) and trainee residents of internal medicine (IMR), family medicine (FMR) and surgery (SR) at tertiary care hospitals of Karachi, Pakistan.

**Methods:**

A validated questionnaire consisting of 21 open ended questions related to diabetes awareness was acquired through a study done at Thomas Jefferson University Hospital, Philadelphia with the permission of primary author.

**Results:**

169 IMR, 27 FMR, 86 SR and 99 RN completed a questionnaire that assessed the knowledge related to different aspects of management of diabetes. The results were further stratified by participant's specialty and level of training. The percentage of knowledge based questions answered correctly was found to be low. The overall mean correct percentage among all the participants was 50% +/- 21. There was no statistical difference in terms of knowledge between IMR & FMR residents (64% +/- 14 vs. 60% +/- 16, p = 0.47) respectively. The total scores of SR and RN were quite low (40% +/- 16 & 31% +/- 15 respectively).SR and RN were found to have profound deficit in both inpatient and outpatient knowledge of diabetes. We did not observe any improvement in **level** of knowledge of FMR & SR with increase in duration of their training (p = 0.47 & 0.80 respectively). In contrast, improvement in the level of knowledge of IMR was observed from first to second year of their training (p = 0.03) with no further improvement thereafter. RN's didn't respond correctly on most of the items related to in-patient management of diabetes (Mean score 40% +/- 20).

**Conclusion:**

As there are no prior studies in our setting evaluating knowledge related to diabetes management among residents and nurses, this study is of paramount importance. Based on these results, considerable knowledge gaps were found among trainee residents and nurses pointing towards need of providing additional education to improve the delivery of diabetes care.

## Background

Since the prevalence of diabetes has been rapidly rising, it has become one of the major public health problems. According to latest data released by an IDF, the worldwide number of people with diabetes has reached 366 million contributing to approximately 4.6 million of deaths [1]. In terms of country wise prevalence if current trends prevail, Pakistan would become tenth leading nation in the world by 2030 comprising of more than 11 million individuals suffering from this burgeoning epidemic [1,2].

Not only the prevalence is high but Pakistanis also develop diabetes and associated complications at a relatively younger age [3] which are the most productive years of one’s life, posing a significant health burden at individual, household, community and health sector levels. This becomes a significant concern in a country like Pakistan where non-availability of national health and scarcity of a health insurance system, the patients have to bear the health cost expenses out of their own pocket. The overall mean economic cost (both direct & indirect) spent by individuals to visit their physicians for outpatient treatment of diabetes is around Rs.2, 070 per visit in Pakistan [4]. The situation becomes gloomier if the inpatient cost of diabetes care is included in the overall expenses. Therefore, the role of a health care professional becomes pivotal in providing safe, effective and evidence based care to these patients to achieve optimal clinical outcomes. Moreover, clinical benefits of adequate glycemic control have been consistently demonstrated in numerous clinical studies [5,6]. Since trainee residents and nurses are involved in provision of primary medical care to the patients where diabetes as a primary diagnosis or is one of the comorbid conditions, it is essential that they should be well equipped in terms of their knowledge to deal with this epidemic effectively. There are several studies that have looked into the issue of diabetes related knowledge among health care professionals [7-9] and in Pakistan such work includes a study done by Shehra et al [10]. However, there has been no exploration of diabetes knowledge of nurses and trainees in Pakistan. Assessment of knowledge among resident trainees is also very important since majority of patients admitted in hospital especially non-medical admissions (surgery, orthopedics etc.) have underlying diabetes which could lead to adverse clinical outcomes if not managed efficiently.

Inadequate understanding and knowledge, related to the time action profile of both premixed and new insulin analogues could also give rise to unfavorable patient outcomes. In addition, health care providers also need to be fully equipped in terms of their knowledge to face medical emergencies related to diabetes since they can occur in any inpatient unit.

The purpose of this study was to assess the knowledge related to both inpatient and out-patient aspects of diabetes management among the trainees in different specialties (Internal Medicine, Family Medicine & Surgery) and nurses and to identify the major areas of deficiency requiring educational reinforcement.

This study is of paramount importance because to date, in our literature search we didn’t find any study evaluating diabetes related knowledge of both trainee residents and nurses in this region. Our goal was to recognize the deficiencies and then to incorporate the themes accordingly in future diabetes education programs. The ultimate aim is to improve the quality of diabetes care in order to reduce the complication rate and health burden of this epidemiologically important chronic disease.

## Research design and methods

This was a Cross sectional, multicenter study. A pre tested validated questionnaire (Appendix) with test- retests reliability of 0.71 consisting of 21 open ended questions related to diabetes awareness. This was acquired from a study done at Thomas Jefferson University Hospital, Philadelphia with the permission of primary author (Rubin et al) [11]. The questions were divided into categories of outpatient (items1-4), medications (items 5-10) and in-patient (items 11- 21).

Furthermore the questionnaire was assessed for content validity by our team of Endocrinologists and nurses with expertise in diabetes care. A 3 member panel (Endocrinologist, Endocrine trainee and one nurse with diabetes teaching background) outside the study team was set up. The questions were assessed qualitatively keeping in view the training system and curriculum of residents and nurses to avoid asking unrelated questions. The content was rated valid by the panel on clarity and relevance and no significant issues were identified.

Furthermore, a pilot of the study was performed in one of the institute initially in separate group consisting of nurses and trainees not included in the final study to assess the level of difficulty of the test.

The questionnaire was administered without any modification at 5 tertiary care university/teaching hospitals in Karachi, Pakistan through non probability purposive sampling method to internal medicine residents (IMR), family medicine residents (FMR), surgery residents (SR) and registered nurses (RN) in wards or at regular meetings after taking their informed consent verbally. Ethical approval was obtained from the Aga Khan University Ethics Review Committee.

Although there were variations in terms of training of residents and nurses among institutes most probably related to limited availability of financial and personnel resources.

The study coordinator visited the selected institutes during working hours and trainee resident and nurses were asked to fill in the forms under his supervision after explaining the purpose of the study in detail and with their consent. Mostly, the participants were approached right after their teaching sessions to catch the maximum number of trainees.

The total time allocated to fill the questionnaire was 15 minutes. The responses were kept anonymous.

### Sample size

The sample size was calculated on an assumption that study sample size of 410 is required to achieve 80% power to detect differences of mean percentage score of 7 among the four groups versus alternative of equal means with a 5% significance level. The standard deviation (SD) between the group is 2.05.The common SD within group is assumed to be 14 [12]. By inflating the sample size to 15% to adjust for nonresponse rate, the final sample size was found to be 472.

## Statistical analysis

Statistical analysis was performed using SPSS software version 16. Frequency and percentages were reported for qualitative variables like correct response of ADA guideline for HbA1c, medication etc. and mean with SD presented for quantitative variables (Specially mean percentage score).

The content analysis strategy was used for coding open ended answers. The reason for using this strategy was the fact that some of the answers were descriptive. Initially coding was formulated by the study team (consisting of Endocrinologist and diabetic nurse) after thoroughly reading all the answers and classified as correct (1) or wrong (0), however, about 5% of the missing or inadequate responses were left uncoded. The resulting coding was again later independently assessed by the study investigators and inter-rater agreement was assessed.

Unanswered answers were counted as wrong (score 0) with an assumption that participants most probably were not aware of the correct answer in view of the open ended nature of the questionnaire. The total score was acquired by adding respective points which were then expressed as mean percentage.

The study participants were characterized using the following variables: professional category (nurse/trainee residents) and years of training. Differences in proportion among specialties were assessed by Pearson Chi-square test however means differences were compared by Analysis of variance (ANOVA) and multiple comparisons within specialty were assessed by Bonferroni test. The survey had a good reliability coefficient (Cronbach α of 0.81). Statistical significance was considered to correspond to a *P* value <0.05.

## Results

Out of the 472 doctors and nurses who were administered the questionnaire 381(80%) responded. The response rate varied between different groups of resident trainees and nurses with rate of 90% from IMR, 83% from FMR, 75% from SR & 73% from RN.

Of the 381 participants, 169 were IMR, 27 were FMR, 86 were SR and 99 were RN (Table 1). Since the responses were similar among the five tertiary care hospitals therefore the data was combined. The mean percentage of correct answers of all the participants was 50% ± 21. The knowledge was found to be highest in residents doing their training in IM with scores of 65% ±14.

**Table 1:** Percentage of Correct Responses to Individual Questionnaire Items According to Group

	**Overall**	**IMR**	**FPR**	**SR**	**RN**	**P value**
	**(n = 381)**	**(n = 169)**	**(n = 27)**	**(n = 86)**	**(n = 99)**	
**Correct Answers (underlined and bolded)**						
**Outpatient setting**						
The ADA HbA1C goal for a patient with diabetes is <7%	239(63%)	146(86%)	24(89%)	53(62%)	16 (16%)	<0.001
According to Adult Treatment Panel III Guidelines, LDL goal in diabetes without known ischemic heart disease should be less than 100	129(34%)	84(50% )	20 (74%)	13(15%)	12(12%)	<0.001
**Medications**						
70/30 is mixture of 70% NPH.	216(57%)	122(72%)	15(56%)	38(44%)	41(41%)	<0.001
70/30 is a mixture of 30% Regular.	234(61%)	132(78%)	18(67%)	38(44 %)	46(47%)	<0.001
70/30 is best administered before meals	339(89%)	161(95%)	24 (89%)	70(81%)	84(85%)	0.004
Rosigliatazone should be discontinued if patient develops congestive heart failure.	221(58%)	146 (86%)	20(74%)	29(34%)	26 (26%)	<0.001
Peak action of regular insulin is 2-4 hours.	163 (43%)	94(56%)	12 (44%)	29( 34%)	28 (28%)	<0.001
**In patient Setting**						
Most important electrolyte to follow for a patient with DKA on insulin drip is Potassium.	324(85%)	165 (98%)	26(96%)	77 (90%)	56(57%)	<0.001
The blood glucose decline per hour on insulin drip should be 50-100mg/dl	121(32%)	86(51%)	5(19%)	17(20%)	13(13%)	<0.001
Oral glucose or juice is the preferred mode of treatment for hypoglycemic alert patient.	277 (73%)	135 (80%)	18 (67%)	54(63%)	70(71%)	0.025
An insulin drip should be discontinued 30-120 minutes following administration of subcutaneous insulin.	128 (34%)	80( 47%)	7 (26%)	19( 22%)	22(22%)	<0.001
For a patient admitted with DKA on an insulin drip IV fluids should be changed to D5 ½ NS at blood glucose level of 200.	170 (45%)	121(72%)	18(67%)	18( 21%)	13(13%)	<0.001

Results are presented as mean ± SD and number (percentages).

No statistical difference was found between the level of knowledge of IMR and FMR (65 ±14 & 60% ± 16 respectively), p = 0.47 (Table 2). Among SR, the overall level of knowledge was found to be significantly low, with mean percentage of 40% ± 16. Like SR, RNs were also found to have significant deficiency in their knowledge with an overall mean percentage score of 31% ± 15.

**Table 2:** Pair wise comparison of correct responses stratified by participant’s category with use of bonferroni test

**Participant’s Category**	**Difference between means**	**95% confidence limits**	**P value**
**IMR versus**			
Family Medicine	4.5	3.5, 12.5	NS
Surgery Residents	24.9	19.8, 30.1	<0.001
Nurses	33.5	28.6, 38.4	<0.001
**Family Medicine versus**			
Surgery Residents	20.4	11.8, 29.0	<0.001
Nurses	29.0	20.5, 37.4	<0.001
**Surgery Residents versus**			
Nurses	8.5	2.8, 14.3	<0.001

The majority of IMR were in their first year of training (n = 59) followed by approximately equal number of participants from second to fourth years. In addition, the level of knowledge showed improvement from their first to second year of training (p = 0.03). Thereafter, their level of knowledge didn’t demonstrate any improvement. On the other hand in comparison to IMR, the level of knowledge of FMR & SR didn’t show improvement with level of experience at all (p = 0.46 & 0.80 respectively).

Some of the questions answered correctly and stratified by type of participant are outlined in table 1.

**Figure 1:**
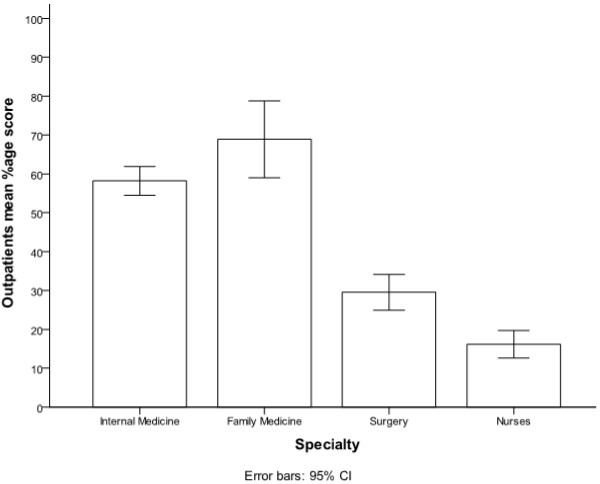
Out-patient mean correct percentage score stratified by participant’s category.

**Figure 2:**
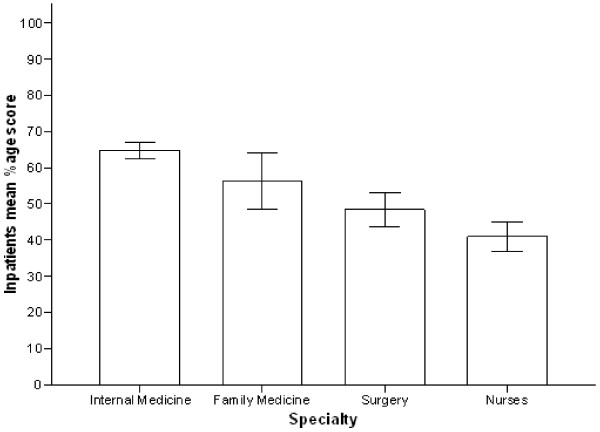
In-patient mean correct percentage score stratified by participant’s category.

**Figure 3:**
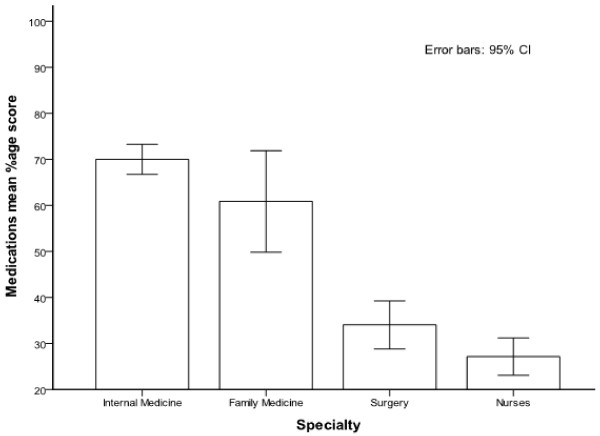
Mean percentage correct score on questions related to diabetic medications stratified by participant’s category.

In the questions addressing out patient management of diabetes, sixty three percent of the participants were aware of the current American Diabetes Association (ADA) recommended goal for HbAIC (nonenzymatic binding of glucose with free amino group of globin chain), but only thirty four percent of the participants knew about the low density lipoprotein cholesterol (LDL) goal for diabetic patients without known ischemic heart disease. After stratifying the results by the participant’s specialty, FMR scored better compared with IMR, 74% vs. 50%, on this particular question (p = 0.018). Two thirds of both IMR and FMR were aware of the criteria for diagnosing diabetes, in sharp contrast to only sixteen percent of SR (p = <0.001). FMR outscored IMR on number of questions related to outpatient management of diabetes but it was not statistically significant (p = 0.12) (Figure 1). Regarding the knowledge about oral anti diabetic agents, 59% of the responders were aware of the fact that metformin (biguanide) is contraindicated in renal insufficiency. Assessment of knowledge about the most commonly used form of insulin in hospitalized patient revealed that less than half of the participants knew about the peak action of regular insulin. Similarly, only 38% of all participants were familiar with the duration of action of various insulin analogs with RN’s displaying the lowest score (2.0%). More than half of the responders were familiar with the exact components of humulin70/30 insulin. IMR scored highest on items pertaining to the knowledge related to oral anti diabetic agents and various insulin preparations compared to FMR but this differences was not statistically significant (p = 0.29). When it came to treatment of mild hypoglycemic episodes RN’s score was similar to that of IMR (71% & 73% respectively). On the other hand only seventeen percent RN’s were aware of the correct treatment for severe hypoglycemia compared to IMR score of 35%. Overall 46% of the participants recognized that continuous intravenous infusion is the ideal mode of treatment for type 1 diabetics undergoing major surgical procedure. No statistical difference was found regarding knowledge of in-patient management of diabetes between IMR and FMR (p = 0.175) (Figure 2). Although the mean percentage score of nurses related to in-patient management was 40% ± 20 considerably better than their out-patient knowledge of 16% ± 18 but still very low in comparison with other published studies. Furthermore, knowledge about insulin and oral anti-diabetic medications was also found to be suboptimal among RN and SR with scores of 27% ± 20 & 34% ± 24 respectively (Figure 3).

### Post hoc statistical power

A post hoc power analysis was conducted using the software package, G*Power version 3.0 (Faul and Erdfelder 1992). The sample size of 381 was used for the statistical power analyses. The effect sizes used for this assessment were as follows: small (0.27), medium (0.6), and large (1.3). The alpha level used for this analysis was 0.05. The post hoc analyses revealed the statistical power for this study was 0.27 for detecting a small effect, whereas the power exceeded .98 for the detection of a moderate to large effect size.

Thus, there was more than adequate power (i.e., power * .80) at the moderate to large effect size level, but less than adequate statistical power at the small effect size level.

## Discussion

Management of Diabetes among other non-communicable diseases has recently drawn substantial attention due to its associated complications and socio-economic impact.

Lack of knowledge among health care providers has been found to be one of the major obstacles in the management of hyperglycemia [13,14]. Among health care providers, our trainee residents and nurses possess a significant role in provision of care to these patients. Before developing a teaching material for educational activities for health professionals, a comprehensive assessment of health professional’s current knowledge must be done. The present study assessed whether our trainee residents and nurses are well-equipped in terms of their knowledge to manage patients with diabetes. Identification of the gaps in the knowledge would help us in highlighting these areas more specifically in future educational programs with an ultimate aim of improving diabetes care delivery in Pakistan.

According to this current study, the overall mean score of the participants was found to be quite low which points towards the inadequacy of diabetes training. The results were even lower to the study, from which the tool was acquired (61%) [11] and other study done in UK [14]. In contrast, the results were better than one of the recent study done in Switzerland (43 ± 22) [12] but the difference could be attributed to the use of a slightly advanced questionnaire. The results were also low in comparison to the previous study investigating the diabetes related knowledge among family physicians in Pakistan (62%) [10]. These results are distressingly low keeping in view the exceedingly high number of diabetics in our country. Since the burden of non-communicable diseases like diabetes not only depends on preventive measures but also on the optimal care provided to existing cases.

The expected increment of knowledge from first to second year of IMR truly reflects impact of good quality postgraduate training. But subsequent lack of improvement consistent with findings from another study [15] points towards the lack of knowledge among senior medicine house staff. Interestingly, one of the intervention studies revealed that even with education the knowledge plateaus at a senior level [16]. In comparison, FMR & SR didn’t show any significant augmentation in their knowledge with their level of experience pointing towards need to add more diabetic sessions and lectures in their curriculum. These findings demonstrate that educational intervention should focus all PGY levels with greater emphasis on reinforcement of the same knowledge through lectures and tutorials. Furthermore, the gap in diabetes related knowledge among SR found in our study and two other previous studies [11,12] is a source of concern since diabetic patients are extremely prone to adverse outcome of surgery if not managed optimally. Moreover, majority of the patients admitted under surgical care have underlying diabetes and evidence suggest that good glycemic control reduces the rate of blood transfusion, the need for dialysis, critical illness polyneuropathy, blood stream infections and hospital stay [17]. One could strongly argue that frequent consultation with diabetologists could resolve this issue. However, reluctance of health care providers to follow the recommendations of endocrine service was one of the most frequent barriers acknowledged in a study to control hyperglycemia in hospitalized patients due to various misconceptions [14]. Hence, to prevail over this clinical inertia, residents including SR need to have basic knowledge concerning management of hyperglycemia. Insulin is identified as one of the highest risk medications, globally and with an advent of new insulin analogues it is observed that most of the trainee residents and nurses are not very well acquainted with these new insulin formulations, so the chances of errors are amplified. Our study clearly proved this and demonstrated that majority of the participants especially nurses were not aware of the time action profile of these new insulin analogues.

The most serious problem recognized in this study was inadequate knowledge of RN regarding management of diabetes. Previous studies have also revealed the deficiency in their knowledge with scores of 41-72% [9,11,12,18,19] but our results are quite inferior to the results of above mentioned studies. Nurses play a major role in the treatment of diabetic patients and should be well equipped to perform recommended standards of care. A Cochrane review revealed that trained nurses can indeed replace physicians in various aspects of diabetes management [20].

### Limitations

One of the limitations of our study might be small sample size of the participants belonging to FMR attributable to paucity of family practice training institutes in our setting. Worldwide, the care of diabetic patients has been moving from specialist to family/general physicians [21] pointing towards the need of more training programs for family medicine specialty in our setting. Undoubtedly family practitioners play fundamental role in promotion of health care and many countries depend on them for provision of affordable care to their patients. This limitation therefore highlights lack of family practice training programs in Pakistan which should be dealt to promote primary health care system in the country.

Another limitation could be the format of questionnaire since open ended questions can create difficulty for test takers and results in larger item non-response [22]. In contrast the chances of bias because of leading questions are reduced in assessment using open ended questionnaire since there are no clues in the questions for the participants.

Although the study tool was not validated on our population, however we performed content validity and agreement between subjects by doing Cronbach α which showed better internal consistency.

On the other hand, the multicenter nature is the major strength of our study maximizing the generalizability of the results. Moderately decent response rate of the participants despite of the use of open ended questionnaire also points towards the fact that trainee residents and nurses are also keen to learn the management of this important disease entity.

## Conclusion

In summary, this study demonstrates considerable gaps in diabetes knowledge of all survey participants especially residents in surgery and nurses which indirectly reflects the quality of care provided to diabetic patients. This led us to conduct the sessions and seminars for residents and nurses to teach them the basic knowledge regarding the management of diabetes focusing mainly on in patient component. Subsequently, standardized protocols and algorithm (both hand written & computerized) have been designed for management of inpatient hyperglycemia based on latest published guidelines for guidance of our trainee residents and nurses. In addition, online education courses for physicians, trainee doctors and nurses is planned to be introduced to fill the gap in diabetes education. There is further need to assess the impact of these interventions on overall knowledge of our trainee residents and nurses in the form of post intervention survey in future.

## Competing interests

The authors declare that they have no competing interests.

## Author’s contribution

AA was the principal author and contributed to the concept, design, literature search and interpretation of data and drafting of manuscript. AJ and LZ contributed to study concept and revision of manuscript and provided important intellectual content. MI contributed to analysis and interpretation of the data. KS contributed to acquisition of data and drafting of manuscript. All authors read and approved the final manuscript.

## Pre-publication history

The pre-publication history for this paper can be accessed here:

http://www.biomedcentral.com/1472-6823/12/18/prepub
